# The In Vitro and In Vivo Anti-Inflammatory Effects of a Phthalimide PPAR-γ Agonist

**DOI:** 10.3390/md15010007

**Published:** 2017-01-04

**Authors:** Mingzhi Su, Jiafu Cao, Jin Huang, Sen Liu, Dong Soon Im, Jin-Wook Yoo, Jee H. Jung

**Affiliations:** College of Pharmacy, Pusan National University, Busan 609-735, Korea; sumingzhi0310@gmail.com (M.S.); caojiafu1985@163.com (J.C.); jellyohuang@gmail.com (J.H.); ls691392008@sina.com (S.L.); imds@pusan.ac.kr (D.S.I.)

**Keywords:** anti-inflammatory effect, PPAR-γ agonist, phthalimide analogue, RAW264.7 macrophage, cytokines, NF-κB pathway, paw edema

## Abstract

Previously, the authors found that 4-hydroxy-2-(4-hydroxyphenethyl) isoindoline-1,3-dione (**PD1**) (a phthalimide analogue) bound to and activated peroxisome proliferator-activated receptor-γ (PPAR-γ). Since PPAR-γ suppresses inflammatory responses, the present study was undertaken to investigate the anti-inflammatory effects of **PD1**. In lipopolysaccharide (LPS)-stimulated murine RAW264.7 macrophages, **PD1** suppressed the inductions of pro-inflammatory factors, including inducible nitric oxide synthase (iNOS), nitric oxide (NO), cyclooxygenase 2 (COX-2), tumor necrosis factor α (TNF-α), interleukin-1β (IL-1β), and interleukin-6 (IL-6). Concomitantly, **PD1** enhanced the expressions of anti-inflammatory factors, such as arginase-1 and interleukin-10 (IL-10), and suppressed LPS-evoked nuclear factor kappa B (NF-κB) p65 subunit phosphorylation in macrophages. In addition, PPAR-γ activated by **PD1** was intensively translocated to the nucleus. These observations suggest that the anti-inflammatory mechanism of **PD1** involves inhibition of the NF-κB pathway. In a subsequent in vivo animal experiment conducted using a carrageenan-induced acute inflammatory rat paw edema model, intraperitoneal injection of **PD1** significantly reduced paw swelling. Histological analysis of rat paw tissue sections revealed less infiltration of immune cells in **PD1**-pretreated animals. These findings suggest that **PD1** be viewed as a lead compound for the development of novel anti-inflammatory therapeutics.

## 1. Introduction

Peroxisome proliferator-activated receptor (PPAR)-γ is a member of the nuclear receptor superfamily, which are activated by ligands, such as endogenous 15-deoxy-Δ^12,14^-prostaglandin J_2_ and insulin-sensitizing thiazolidinediones [1]. Activated PPAR-γ translocates to the cell nucleus and binds to peroxisome proliferator response element (PPRE) on target DNA to regulate gene transcription. PPAR-γ is predominantly present in adipose tissue, colon, skeletal muscle, endothelium, macrophages, and monocytes, and plays important roles in the regulation of insulin sensitivity, lipid metabolism, adipogenesis, and glucose homeostasis [2]. PPAR-γ is also involved in the regulations of immune and inflammatory responses [3,4]. Many inflammatory diseases stem from the activation of macrophages, and it is known that classically activated M1 macrophages induced by infection, tissue damage, or exposure to endotoxins (i.e., lipopolysaccharide (LPS)) secrete large amounts of pro-inflammatory mediators, such as nitric oxide (NO), inducible nitric oxide synthase (iNOS), cyclooxygenase 2 (COX-2), cytokines interleukin-1β (IL-1β), interleukin-6 (IL-6), and tumor necrosis factor α (TNF-α), whereas activated M2 macrophages secrete Transforming growth factor beta 1 (TGF-β1), IL-10, and arginase-1, and thus play important anti-inflammatory and tissue restorative roles. A recent study revealed that the anti-inflammatory effects of PPAR-γ are due to its modulation of macrophage activation, which is a prerequisite for the optimal induction of the M2 macrophage phenotype by interleukin-4 [4]. It is also known that PPAR-γ activation leads to the repression of pro-inflammatory genes, such as iNOS, COX-2, and IL-6, and inhibits the expressions of cytokines and acute phase proteins by inhibiting the nuclear factor kappa B (NF-κB) pathway [4,5,6,7,8].

Over the past decade, it has been demonstrated that PPAR-γ agonists are potentially beneficial for treating several inflammatory diseases. Rosiglitazone, which is used clinically to treat type II diabetes mellitus, has been reported to inhibit the expressions of inflammatory cytokines during LPS-induced acute kidney injury [9], and to have anti-inflammatory effects in carrageenan-induced paw edema and carrageenan-induced pleurisy [10]. In addition, rosiglitazone and other PPAR-γ agonists were found to be effective in a murine airway-inflammation model by reducing airway hyper-responsiveness and mucus production [11], and to suppress neutrophilia and chemoattractant production, thus inhibiting inflammatory response [12]. PPAR-γ agonists have also been reported to be effective in models of myocarditis and intestinal inflammation [13,14]. For example, pioglitazone and telmisartan both improved myocardial injury in diabetes by inhibiting receptor of advanced glycation end products (RAGE)-mediated oxidative stress and inflammation [15,16]. Furthermore, our group previously reported that a jasmonate analogue designed and synthesized as a PPAR-γ agonist exerted anti-inflammatory effects in murine colitis [17,18].

**Figure 1:**
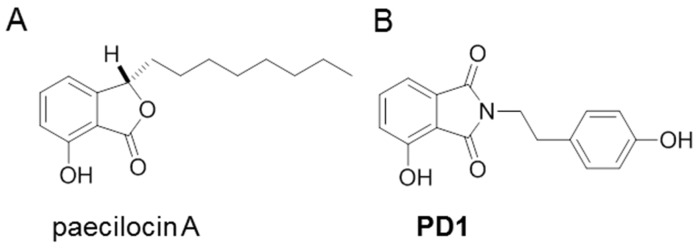
Chemical structures of paecilocin A (**A**) and **PD1** (4-hydroxy-2-(4-hydroxyphenethyl) isoindoline-1,3-dione) (**B**).

In a previous study, we isolated a new marine natural product paecilocin A (Figure 1A) from the jellyfish-derived fungus *Paecilomyces variotii* with good antibacterial activity [19]. In a subsequent screening for PPAR-γ competitive binding activity, synthetic paecilocin A analogues showed PPAR-γ binding activity comparable to that of rosiglitazone [20]. Thereafter, we designed and synthesized a series of *N*-substituted phthalimide analogues based on the pharmacophore study of paecilocin A and synthetic analogues. One of the *N*-substituted phthalimide analogues, 4-hydroxy-2-(4-hydroxyphenethyl) isoindoline-1,3-dione (**PD1**, Figure 1B), was found to exhibit significant PPAR-γ agonistic activity in Ac2F murine liver cells and in HepG2 human liver cells [21,22].

In this study, we investigated the anti-inflammatory effects of **PD1** using LPS-activated RAW264.7 murine macrophages. The productions of pro-inflammatory and anti-inflammatory mediators were examined at the cellular level, and an in vivo study was conducted to examine the anti-inflammatory activity of **PD1** using a carrageenan-induced acute inflammatory paw edema model. In addition, we provide a discussion of the anti-inflammatory mechanism of **PD1** in LPS-induced RAW264.7 macrophages.

## 2. Results and Discussion

### 2.1. **PD1** Promoted PPAR-γ Translocation to Cell Nuclei

**Figure 2:**
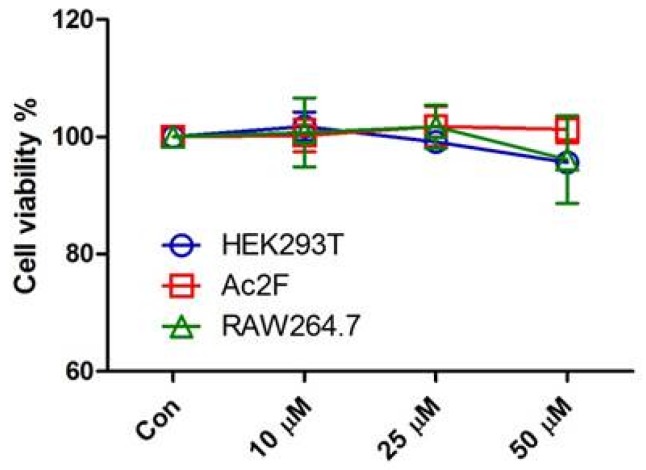
Cytotoxicity evaluation of **PD1** (10, 25, or 50 μM) on human embryonic kidney cells (HEK293T), Ac2F rat liver cells, and RAW264.7 macrophages at 24 h.

Prior to in vitro anti-inflammatory evaluation of **PD1**, the potential toxicity of **PD1** was tested in human embryonic kidney cells (HEK293T), rat liver Ac2F cells, and RAW264.7 murine macrophages. **PD1** was found to be non-cytotoxic to any of these cell lines after exposure to concentrations up to 50 μM for 24 h (Figure 2).

**Figure 3:**
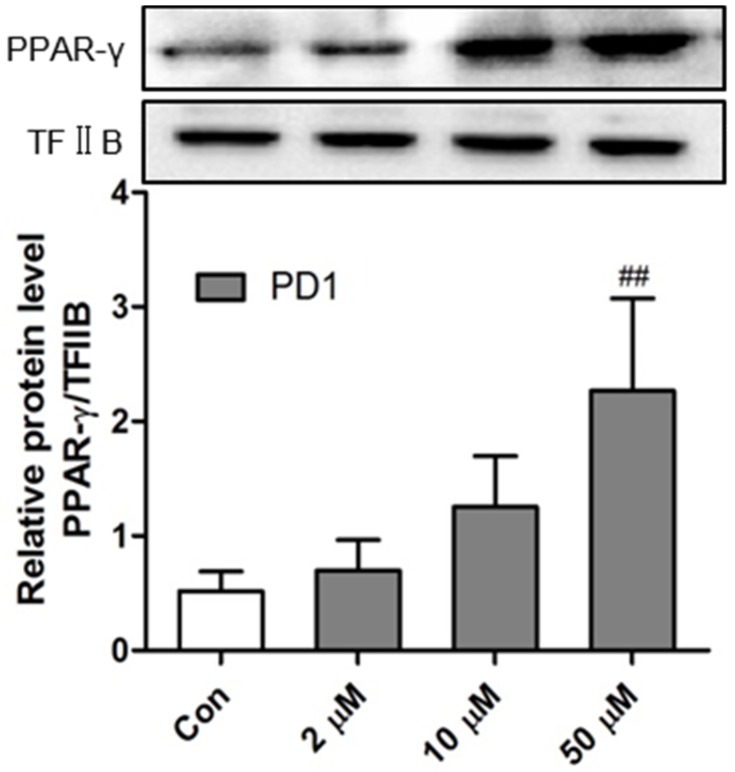
Endonuclear peroxisome proliferator-activated receptor-γ (PPAR-γ) protein levels in RAW264.7 macrophages treated with **PD1** for 24 h as determined by Western blotting. Nuclear levels of transcription Factor II B (TF II B) were used for reference purposes. The results shown are representative of three independent experiments. ^##^ *p* < 0.01 vs. untreated controls.

After ligand binding, PPAR-γ translocates to the nucleus and modulates gene transcription and expression. In a previous study, the PPAR-γ agonistic activity of **PD1** was examined by luciferase assay using PPRE-luciferase reporter plasmid [21,22]. In the present study, we assessed levels of **PD1**-activated PPAR-γ proteins translocated to the cell nuclei of RAW264.7 cells by Western blot. As shown in Figure 3, endonuclear PPAR-γ protein levels were significantly and concentration-dependently enhanced by **PD1** treatment. PPAR-γ expressed in inflammatory cells can regulate metabolic and inflammatory processes [23]. Thus, we hypothesized that endonuclear PPAR-γ activated by **PD1** could initiate a series of responses that regulate inflammatory and/or resolution processes.

### 2.2. Decreased Expression of Pro-Inflammatory Factors by **PD1**

**Figure 4:**
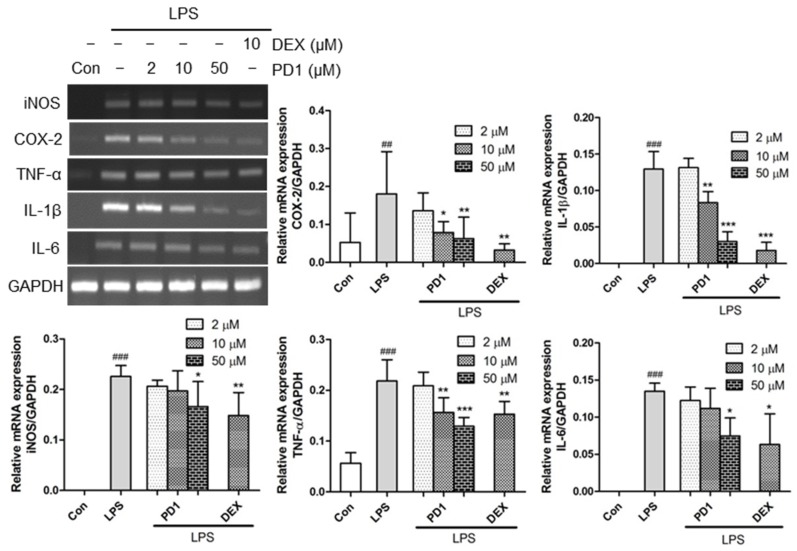
Reverse transcriptase-PCR (RT-PCR) results of pro-inflammatory genes. RAW264.7 cells were pre-treated with the indicated concentrations of **PD1** or dexamethasone (DEX) for 1h, and then treated with lipopolysaccharide (LPS) (10 ng/mL, 1 h). Glyceraldehyde 3-phosphate dehydrogenase (GAPDH) was used as the internal reference. The results shown are representative of three independent experiments. ^##^ *p* < 0.01, ^###^ *p* < 0.001 vs. untreated controls. * *p* < 0.05, ** *p* < 0.01, and *** *p* < 0.001 vs. LPS-treated cells.

In order to elucidate the role of the **PD1** in inflammation, we examined the transcription levels of pro-inflammatory factors. Macrophages stimulated with LPS showed substantial increases in the levels of pro-inflammatory factors. To investigate the anti-inflammatory effect of **PD1**, the mRNA levels of specific genes, such as iNOS, COX-2, TNF-α, IL-1β, and IL-6 in RAW264.7 cells, were assessed using reverse transcriptase-PCR (RT-PCR). It was found that the mRNA levels of pro-inflammatory mediators were markedly increased when murine macrophages RAW264.7 were exposed to LPS for 1 h, and that these increases were significantly and concentration-dependently diminished by pre-treating cells with **PD1** (Figure 4). Notably, at a concentration of 50 μM **PD1** significantly inhibited the mRNA levels of iNOS, COX-2, TNF-α, IL-1β, and IL-6 to extents comparable to those of 10 μM dexamethasone.

**Figure 5:**
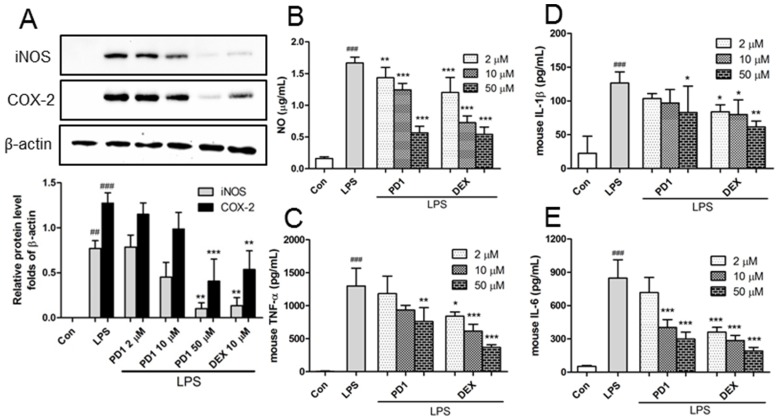
Effect of **PD1** on the inductions and secretions of inducible nitric oxide synthase (iNOS) and cyclooxygenase 2 (COX-2) (**A**); nitric oxide (NO) (**B**); tumor necrosis factor α (TNF-α) (**C**); interleukin -1β (IL-1β) (**D**); and interleukin-6 (IL-6) (**E**). RAW264.7 macrophages were pre-treated with the indicated concentrations of **PD1** or dexamethasone (DEX) for 1 h and then with LPS (1 μg/mL, 24 h), and iNOS and COX-2 levels were determined by Western blot. RAW264.7 macrophages were pre-treated with **PD1** or dexamethasone (DEX) for 1 h and then with LPS (25 ng/mL, 24 h), and NO concentration in medium were determined by Griess method, and the expression levels of IL-1β, IL-6, and TNF-α in medium were determined by enzyme-linked immunosorbent assay (ELISA). In the case of TNF-α, cells were pre-treated with **PD1** or dexamethasone for 1 h and then with LPS (25 ng/mL, 3 h). The results shown are representative of three independent experiments. ^##^ *p* < 0.01, ^###^ *p* < 0.001 vs. untreated controls. * *p* < 0.05, ** *p* < 0.01, and *** *p* < 0.001 vs. LPS-treated cells.

In addition, the protein levels of the pro-inflammatory factors iNOS and COX-2 were analyzed by Western blot and enzyme-linked immunosorbent assay (ELISA). In LPS-induced RAW264.7 cells, both were inhibited by **PD1** (Figure 5A). Since iNOS is a family of enzymes that catalyze NO production from l-arginine, we supposed reductions in iNOS expression by **PD1** should lead to decreased NO production in macrophages, and thus we measured NO amounts in RAW264.7 cell supernatants using Griess reagent. NO levels were found to be significantly increased by LPS (Figure 5B), but these increases were significantly and concentration-dependently inhibited by **PD1** pre-treatment. For example, after treatment with 50 μM **PD1**, LPS-induced NO increases were inhibited by 66%. Furthermore, **PD1** pre-treatment reduced the LPS-induced secretions of TNF-α, IL-1β, and IL-6 (Figure 5C–E). This result is consistent with those of other studies, which reported that treatment of monocytes or macrophages with high concentrations of PPAR-γ agonists reduced the secretions of pro-inflammatory cytokines by inhibiting macrophage activation [10].

### 2.3. **PD1** Enhanced the Expressions of Anti-Inflammatory Factors

**Figure 6:**
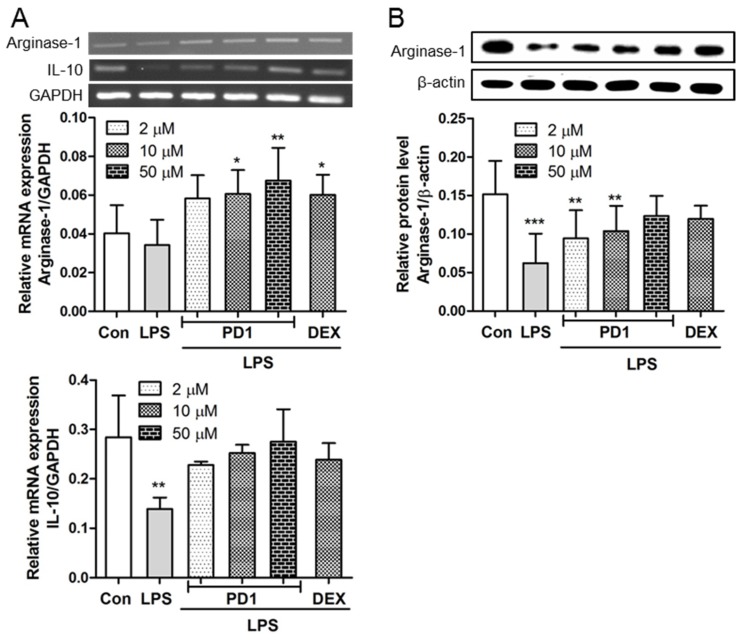
RT-PCR results for anti-inflammatory genes (**A**) and Western blot results for the protein expression of arginase-1 (**B**). RAW264.7 cells pre-treated with the indicated concentrations **PD1** or dexamethasone (DEX) for 1 h and then treated with LPS (10 ng/mL, 1 h) for RT-PCR, or with LPS (1 μg/mL, 24 h) for Western blot. GAPDH and β-actin were used as internal references, respectively. The results shown are representative of three independent experiments. * *p* < 0.05, ** *p* < 0.01, and *** *p* < 0.001 vs. untreated controls.

Both iNOS and arginase-1 are required for the metabolism of l-arginine. Arginase-1 affects inflammatory responses through l-arginine metabolism, which generates proline and polyamines, and ultimately enhances collagen synthesis, cell proliferation, tissue repair, and wound healing [24]. The anti-inflammatory factors arginase-1 was assessed in both mRNA (Figure 6A) and protein levels (Figure 6B). After stimulating RAW264.7 macrophages with LPS, the expression of arginase-1 decreased, but this reduction was almost totally recovered by **PD1** pre-treatment. Gao et al. showed activation of the signal transducer and activator of transcription 6 (STAT6) signaling pathway promotes PPAR-γ and arginase-1 expressions simultaneously, and that PPAR-γ synergistically augments the expression of arginase-1 [25].

Furthermore, the transcriptional level of IL-10 was reduced by LPS treatment in RAW264.7 macrophages, and as was observed for arginase-1, this reduction was diminished by **PD1** pre-treatment (Figure 6A). By attenuating the activations of immune cells, IL-10 acts as a potent antagonist of pro-inflammatory cytokines. The principal function of IL-10 appears to be the suppression and ultimately the termination of inflammatory responses [26]. In fact, IL-10 has the ability to suppress the expressions of several pro-inflammatory factors, including interferon-γ (IFN-γ), IL-1β, IL-6, and TNF-α in macrophages and lymphocytes, and to inhibit the production of prostaglandin E_2_ (PGE_2_) by reducing COX-2 expression [27,28]. Yang et al. pointed out that the transcriptional activity of IL-10 is strongly related to PPAR-γ expression [29]. Furthermore, PPAR-γ agonists have been shown to induce IL-10 production and enhance protective effects in an asthma model [30]. Our results show **PD1** exerts an anti-inflammatory effect by inhibiting the pro-inflammatory mediators (NO, iNOS, COX-2, IL-1β, IL-6, and TNF-α) and promoting the expressions of anti-inflammatory mediators (arginase-1 and IL-10) in RAW264.7 cells.

### 2.4. Modulation of the NF-κB Pathway by **PD1**

**Figure 7:**
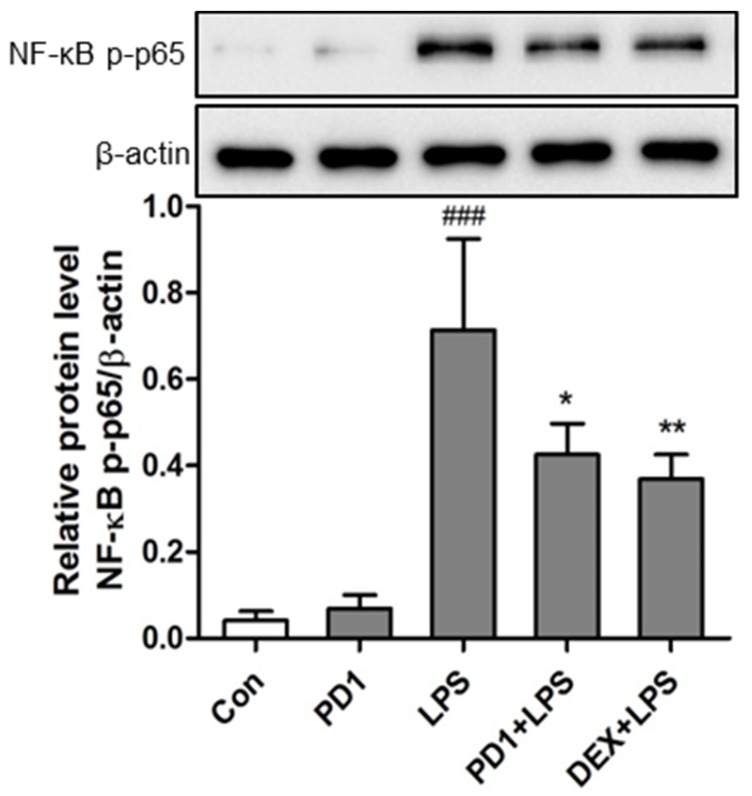
Phosphorylated nuclear factor kappa B (NF-κB) p65 levels in RAW264.7 murine macrophages were determined by Western blot using β-actin as the internal control. Cells were pretreated with **PD1** (50 μM) or dexamethasone (DEX, 50 μM) for 30 min, and stimulated with 20 ng/mL LPS for 15 min. ^###^ *p* < 0.001 vs. untreated controls. * *p* < 0.05 and ** *p* < 0.01 vs. LPS-treated cells.

Some authors have suggested that PPAR-γ interferes with several transcription factors involved in pro-inflammatory signal pathways, such as activator protein-1 (AP-1), signal transducers and activators of transcription 1 (STAT-1), and nuclear factor of activated T cells (NFAT) [23]. However, others have shown that PPAR-γ suppresses the pro-inflammatory process by repressing NF-κB activity [5]. To investigate the mechanism responsible for the effect of **PD1** on LPS-activated NF-κB signaling, we examined NF-κB p65 phosphorylation levels in RAW264.7 macrophages. As was expected, the protein levels of phosphorylated p65 subunits were significantly increased in cells after LPS (20 ng/mL) treatment for 15 min (Figure 7), indicating that LPS exposure promotes activation of the NF-κB pathway. However, **PD1** pre-treatment significantly reduced NF-κB p65 phosphorylation with a potency comparable to that of dexamethasone at the same concentration (50 μM). It was previously supposed PPAR-γ might antagonize NF-κB activity via some protein–protein interaction [10,18]. Our findings suggest that **PD1** inhibits the inflammatory process by suppressing the NF-κB pathway.

### 2.5. Anti-Inflammatory Effect of **PD1** in a Carrageenan-Induced Paw Edema Model

**Figure 8:**
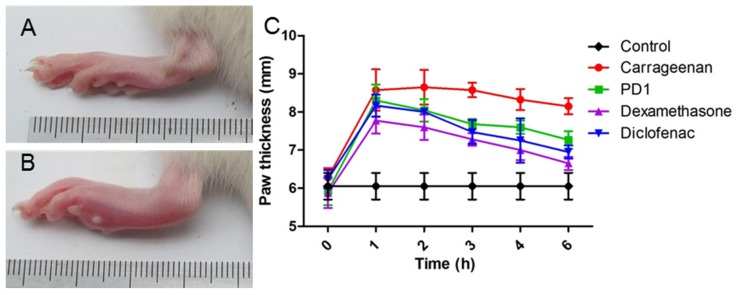
Changes in the paw thicknesses (mm) of Wistar rats. Photographs showing a representative paw before (**A**) and after (**B**) carrageenan injection. Changes in paw thickness after pre-treatment with **PD1**, dexamethasone, or diclofenac (50, 20, and 20 mg/kg, respectively) (**C**). Results are presented as means ± SDs of *n* = 6 rats/group. Control: phosphate buffered saline (PBS)-injected; Carrageenan: carrageenan-injected; **PD1**: **PD1**-pretreated; Dexamethasone: dexamethasone-pretreated; Diclofenac: diclofenac-pretreated.

The anti-inflammatory effects of **PD1** were evaluated in vivo using a carrageenan-induced paw edema model, as previously described [31]. Injection of 1% carrageenan phosphate buffered saline (PBS) solution 0.1 mL into a hind paw of Wistar rats resulted in edema, which was assessed by measuring paw thicknesses (Figure 8). Carrageenan was found to have a maximum effect at 2 h after injection when 37.8% paw thickening was observed. Swelling was sustained for 3 h and slowly decreased to 29.9% at 6 h post-injection. Intraperitoneal injection of **PD1** (50 mg/kg) 1 h before carrageenan injection suppressed paw edema. The mean paw thickening was 40.1% in 1 h, 35.7% at 2 h, 29.6% at 3 h, and 22.6% at 6 h. This suppressive efficacy of **PD1** was comparable to that of diclofenac (20 mg/kg), but lower than that of dexamethasone (20 mg/kg).

**Figure 9:**
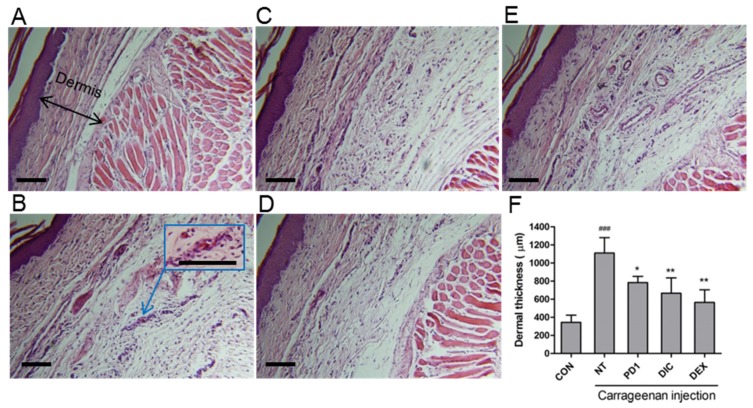
Histological examination of paw tissue sections 3 h after carrageenan injection. (**A**) normal control; (**B**) carrageenan control; and after (**C**) pretreatment with **PD1**; (**D**) dexamethasone; or (**E**) diclofenac. High levels of accumulations of immune cells and fluid collection were observed in carrageenan-treated rats (arrowed in **B**); (**F**) dermal thicknesses were measured in a quantitative manner using a caliper. Control (CON), PBS-injected; non-treatment (NT), carrageenan-injected; **PD1**, **PD1**-pretreated; DIC, diclofenac-pretreated; DEX, dexamethasone-pretreated. ^###^ *p* < 0.001 vs. CON; * *p* < 0.05 and ** *p* < 0.01 vs. NT. Scale bar: 100 μm.

Microscopic photographs of hematoxylin and eosin stained normal paw tissue are shown in Figure 9A. High levels of infiltration-related damage, due to accumulations of immune cells and fluid collection, were observed in carrageenan-treated rats (arrowed in Figure 9B). However, **PD1** (Figure 9C), dexamethasone (Figure 9D), and diclofenac pretreated (Figure 9E) rats showed only moderate infiltration damage. The anti-inflammatory of **PD1** pretreatment was comparable to that of the positive controls diclofenac and dexamethasone. Notable dermal thickening was also observed in carrageenan-treated rats due to fluid collection. Quantitative analysis of dermal thicknesses showed that the dermises of carrageenan treated animals were significantly thickened by fluid infiltration (Figure 9F). **PD1**, dexamethasone, and diclofenac pretreated animals showed similar reductions in carrageenan-induced thickening.

Cytokines TNF-α, IL-1β, and IL-6 are small regulatory proteins produced by activated immune cells that associate with cellular injuries and signs of inflammation and contribute to fever, acute phase protein release, vascular permeability, and inflammatory cell recruitment [32]. Considering the importance of the role played by cytokines in inflammatory response, we evaluated the effect of **PD1** in a carrageenan-induced acute inflammation model to assess the effect of the PPAR-γ agonist **PD1**. Inflammatory symptoms as assessed by paw thicknesses, tissue damage, fluid collection, and immune cell infiltration were found to be alleviated by **PD1**. These findings indicate the anti-inflammatory effects of **PD1** are due to its ability to reduce the productions of pro-inflammatory mediators. 

## 3. Materials and Methods

### 3.1. Materials

Phthalimide analogue **PD1**, 4-hydroxy-2-(4-hydroxyphenethyl)isoindoline-1,3-dione, was synthesized by our group [21]. Dimethylsulfoxide (DMSO), diclofenac, dexamethasone, lipopolysaccharide, Griess reagent, and carrageenan were purchased from Sigma-Aldrich (St. Louis, MO, USA). Primers for RT-PCR experiment were synthesized by BIONEER (Daejeon, Korea).

### 3.2. Cell Culture and Cell Viability

RAW264.7 murine macrophages were purchased from the Korean Cell Line Bank (KCLB^®^, Seoul, Korea), and rat liver Ac2F cells and human embryonic kidney 293T cells (HEK 293T) were obtained from the American Type Culture Collection (ATCC, Rockville, MD, USA). Cells were cultured at 37 °C in a 5% CO_2_ humidified incubator and maintained in high glucose Dulbecco’s Modified Eagle Medium (DMEM, Nissui, Tokyo, Japan) containing 100 mg/mL streptomycin, 2.5 mg/L amphotericin B and 10% heat-inactivated fetal bovine serum (FBS). Cells were seeded in a 96-well culture plates and cultured for 12 h and then treated with various concentrations of **PD1** for 24 h. Cell viabilities were evaluated using water soluble tetrazolium (WST) reagent (EZ-CyTox, Daeil Lab Service Co., Ltd., Seoul, Korea), which was added to each well (10 μL) and incubated at 37 °C for 1 h. Absorbances were read using a iMark Microplate Absorbance Reader (Bio-Rad Laboratories, Hercules, CA, USA) at a wavelength of 450 nm.

### 3.3. Reverse Transcriptase-PCR (RT-PCR)

To determine the expressions of inflammatory markers in RAW264.7 cells by RT-PCR, total RNA was isolated using Trizol reagent (Invitrogen, Carlsbad, CA, USA), and first strand cDNA was synthesized using total RNA and the M-MLV cDNA Synthesis kit (Enzynomics, Daejeon, Korea). cDNA products and primers for each gene were used for PCR with AccuPower^®^ PCR PreMix (BIONEER, Seoul, Korea) in a BIO-RAD T100™ Thermal Cycler PCR unit (Hercules, CA, USA). Specific primers for iNOS (sense 5′-ACC TAC CAC ACC CGA GAT GGC CAG-3′, antisense 5′-AGG ATG TCC TGA ACA TAG ACC TTG GG-3′), COX-2 (sense 5′-CCG TGG GGA ATG TAT GAG CA-3′, antisense 5′-CCA GGT CCT CGC TTA TGA TCT G-3′), TNF-α (sense 5′-AGCACAGAAAGCATGATCCG-3′, antisense 5′-GTTTGCTACGACGTGGGCTA-3′), IL-1β (sense 5′-GGA GAA GCT GTG GCA GCT A-3′, antisense 5′-GCT GAT GTA CCA GTT GGG GA-3′), IL-6 (sense 5′-TGG GAA ATCGTG GAA ATG AG-3′, antisense 5′-GAA GGA CTC TGG CTT TGT CT-3′), and GAPDH (sense 5′-TTC ACC ACC ATG GAG AAG GC-3′, antisense 5′-GGC ATG GAC TGT GGT CAT GA-3′) were used to amplify gene fragments. PCR was performed over 24 cycles of denaturation at 95 °C for 30 s, annealing at 60 °C for 30 s, and elongation at 72 °C for 30 s. Arginase-1 (sense 5′-GTG AAG AAC CCA CGG TCT GT-3′, antisense 5′-CTG GTT GTC GGG GAG TGT T-3′) and IL-10 (sense 5′-CCA AGC CTT ATC GGA AAT GA-3′, antisense 5′-TTT TCA CAG GGG AGA AAT CG-3′) were used to amplify gene fragments. PCR was performed over 24 cycles of denaturation at 95 °C for 30 s and annealing at 55 °C for 30 s. Aliquots (6 μL) were electrophoresed in 1.2% agarose gels and stained with ethidium bromide [33].

### 3.4. Western Blot

Cells were harvested and suspended in lysis buffer containing protease and phosphatase inhibitor cocktails. Nuclear protein extraction was performed using NE-PER^®^ nuclear and cytoplasmic extraction reagents (Thermo Scientific, Rockford, IL, USA). Concentrations of proteins were determined using a BCA protein assay (Thermo Scientific, Rockford, IL, USA). Equal amounts of proteins were resolved by 8% SDS-polyacrylamide gel electrophoresis and electrophoretically transferred to polyvinylidene difluoride (PVDF) membranes, which were then blocked in Tris-buffered saline containing 0.1% Tween 20 (TBS-T) and 5% skimmed milk for 1 h at room temperature, and then incubated with specific primary antibodies recognizing COX-2, iNOS, arginase -1, PPAR-γ, and NF-κB p-p65 (Cell Signaling Technology, Danvers, MA, USA) overnight at 4 °C. Anti-rabbit horseradish-linked IgG was used as the secondary antibody. Signals were developed using the ChemiDoc™ Touch Imaging System (Bio-Rad Laboratories, Hercules, CA, USA).

### 3.5. Production Levels of NO and Cytokines

RAW264.7 macrophages (1 × 10^4^ cells/well) were seeded in a 96-well culture plate and cultured for 12 h. Cells were pre-treated with various concentrations of **PD1** or dexamethasone for 1 h and then co-incubated with 25 ng/mL of LPS for 24 h. NO concentrations in medium were determined using a Griess assay. Griess reagent (80 μL) was added to media supernatants (80 μL) and then incubated at 37 °C for 15 min in the dark. Absorbance was measured at 520 nm. NO concentrations were calculated using 0–100 μM sodium nitrite standards. TNF-α, IL-1β, and IL-6 expression levels in culture medium were quantified using a sandwich-type ELISA kits (Biolegend, San Diego, CA, USA).

### 3.6. Animals

Male Wistar rats (130–150 g, 5-weeks old) were purchased from Daehan Biolink (DBL, Seoul, Korea) and housed under standard laboratory conditions (22 °C ± 2 °C under a 12 h light/dark schedule) with free access to food and water. Prior to use, animals were allowed 1 week to acclimatize. The animal protocol used in this study was reviewed and approved beforehand by the Pusan National University–Institutional Animal Care and Use Committee (PNU–IACUC) with respect to the ethicality of procedures and animal care.

### 3.7. Carrageenan-Induced Rat Paw Edema Assay

The carrageenan-induced hind paw edema was used to assess anti-inflammatory activity, as previously described [31]. Animals were divided into four groups (*n* = 6/group, three of which were used for histological analysis). **PD1** (50 mg/kg), dexamethasone (20 mg/kg), and diclofenac (20 mg/kg) were dissolved in PBS containing 10% DMSO and administered once by intraperitoneal injection. Vehicle controls were injected with vehicle alone. Thirty minutes post-injection, paw edema was induced by a subplantar injection of 0.1 mL of 1% freshly prepared carrageenan suspension in PBS into the left hind paw of each rat. Right hind paws were injected with 0.1 mL of PBS. To gauge the extents of inflammation, paw thicknesses were measured before (0 h) and at 1, 2, 3, 4, and 6 h after carrageenan injection using a digital vernier caliper (Mitotoyu, Series 500, Tokyo, Japan).

### 3.8. Histology

Three animals from each group were euthanized 3 h after carrageenan injection. Paw samples were taken for histological examination. Sectioned tissues were stained with hematoxylin and eosin and viewed under bright field microscope (OLYMPUS, BX53F, Tokyo, Japan).

### 3.9. Statistics

The significances of intergroup differences were determined by ANOVA. Results are expressed as the means ± SDs of indicated numbers of independent experiments. Statistical significance was accepted for *p*-values of < 0.05.

## 4. Conclusions

The present study shows that the PPAR-γ agonist **PD1** has substantial in vitro and in vivo anti-inflammatory activities. The mechanism underlying these anti-inflammatory effects appears to involve the activation of PPAR-γ and the suppression of NF-κB. In turn, the activation of PPAR-γ by **PD1** reduced the expressions of pro-inflammatory mediators, that is, iNOS, NO, COX-2, TNF-α, IL-1β, and IL-6, by inhibiting NF-κB, and concomitantly increased the expressions of anti-inflammatory mediators, such as arginase-1 and IL-10. Furthermore, in our rat paw edema model, **PD1** substantially reduced acute inflammation and attenuated immune cell infiltration. These findings demonstrate that **PD1** has potential use as a lead compound by those developing therapeutic agents for the treatment of acute inflammation.
